# Routine HIV Screening During Intake Medical Evaluation at a County Jail — Fulton County, Georgia, 2011–2012

**Published:** 2013-06-21

Fulton County Jail (FCJ) in Atlanta, Georgia, is one of the 50 largest jails in the nation, with an average daily census of 2,269 detainees (1). During January 1, 2011–March 15, 2012, FCJ implemented a demonstration project to integrate routine rapid human immunodeficiency virus (HIV) screening into the medical intake process. This report summarizes the results. Nearly 59% of persons booked (22,920 of 39,073) received an intake medical evaluation, and voluntary oral fluid HIV rapid screening was offered, except to those who disclosed a previous HIV diagnosis (473 [2.1%]) or were not able to provide consent. An HIV test was offered on 18,869 visits, and 12,141 HIV tests were conducted. All persons with a reactive result (120 [1.0%]) underwent confirmatory HIV testing unless they subsequently disclosed a previous HIV diagnosis. This project identified 52 persons with newly diagnosed HIV infection; 48 by rapid testing (0.4% of those tested) during the study period. All received medical care in the facility and referral for community services on release. Without this HIV screening project, these persons likely would have been diagnosed later in the course of their infection, resulting in delayed access to care and treatment, and possible transmission of HIV to their partners. Linkage to community services is critical, and coordination with the public health system and community-based organizations are essential to ensure access to HIV care and retention in treatment for persons with HIV released from jail.

Jail nursing staff provided opt-out, rapid HIV testing by oral mucosal swab as a standard component of medical services 24 hours a day, 7 days a week, except for a 6-week period (June 30–August 15, 2011) after a change in the contractor providing medical services for FCJ, when only limited, conventional HIV testing was available. A total of 39,073 bookings into FCJ occurred during the HIV screening project period, representing 31,314 persons, because some persons (17.0%) were booked more than once during this period. A newly diagnosed case of HIV was defined by Western blot laboratory confirmation of infection in a person with no record of a previous HIV diagnosis in either the Fulton County or Georgia Department of Public Health HIV surveillance registry or a FCJ medical chart. The cost per new diagnosis in this program was approximately $7,000 (2). Before implementing the demonstration project, syphilis was the only sexually transmitted infection routinely screened for during the intake medical evaluation, and HIV testing was only available on an opt-in basis. Detainees who requested an HIV test had an additional tube of blood drawn and sent to an outside laboratory for enzyme immunoassay (EIA) with reflex Western blot confirmatory testing, with results available within 14 days. During a 3-month period in 2010, when testing required phlebotomy and conventional testing, the acceptance of HIV screening was 43.2% (2,253 of 5,218 jail entrants). During this demonstration project, acceptance of HIV testing increased by 49%, to 64.3% (12,141 of 18,869), when routine rapid HIV testing of oral fluid, rather than conventional testing, was offered (p<0.001).

FCJ recorded HIV test data to determine the number and characteristics of persons newly diagnosed with HIV from January 1, 2011, through March 15, 2012. Two of 52 newly diagnosed persons received venipuncture alone in early August 2011, when rapid testing was unavailable, and two of the positive oral mucosal swabs occurred on December 29, 2010 (Table). All 52 new diagnoses were among non-Hispanic black men (n = 47) and women (n = 5). Among men with a newly diagnosed HIV infection, 38% (n = 18) reported ever having sex with men. Approximately 69% (36 of 52) of newly diagnosed persons reported a previous HIV test (range: 4 months–4 years earlier); 42% (22 of 52) reported a negative HIV test result in the past 2 calendar years, and one person had a negative HIV test result at FCJ admission 4 months earlier. Obtaining a CD4 count often was delayed until a formal medical evaluation was conducted up to 2 weeks after intake, so only 42% (22 of 52) of the cases had a CD4 cell count recorded in the medical record, with a mean of 372 cells/mm^3^. Of persons newly diagnosed with HIV, approximately 17% (nine) were detained for ≤48 hours, and nearly 58% (30) were detained ≤14 days.

## Reported

*Anne C. Spaulding, MD, Chava J. Bowden, Bryan I. Kim, DVM, Mario C. Mann, MBA, Lesley Miller, MD, Genetha R. Mustaafaa, MA, Ryan P. Kyle, MPH, Michelle Leon, Mary V. Mbaba, MPH, Lauren C. Messina, MSPH, Emory Univ, Atlanta, Georgia. Sharne Hampton, MD, Corizon Correctional Healthcare, Brentwood, Tennessee. Robin MacGowan, MPH, Laurie Reid, MA, Andrew Margolis, MPH, Lisa Belcher, PhD, National Center for HIV/AIDS, Viral Hepatitis, STD, and TB Prevention, CDC.* ***Corresponding contributor:*** *Anne C. Spaulding, aspauld@emory.edu, 404-727-3369.*

## Editorial Note

Diagnosis of HIV infection is the first step in accessing care and treatment services and preventing future cases of HIV infection. Providing HIV screening during the medical intake process in detention facilities can identify cases of HIV infection among persons who have not been diagnosed through other clinical or nonclinical community-based HIV testing (3). Incorporating routine HIV screening into the FCJ medical intake process resulted in 52 persons being newly diagnosed with HIV infection during the 15-month period. Consistent with findings from a previous jail study (3), available first CD4 counts were high (mean: 372 cells/mm^3^), indicating diagnosis relatively early in the course of disease. Without this HIV screening project, these persons would likely have been diagnosed later in the course of their infection, resulting in delayed access to care and treatment, and possible transmission of HIV to their partners.

HIV testing is a critical component of the National HIV/AIDS Strategy (4), and an estimated 49% of new infections each year are acquired from persons who are unaware of their infection (5). To prevent new cases of HIV infection in the United States, persons at-risk for HIV infection should be screened for HIV at least annually (6). However, approximately 58% of detainees at FCJ with newly diagnosed HIV infection had not been tested in the past 2 calendar years; only 15% (eight of 52) reported being tested in the past calendar year, and 31% (16 of 52) stated that they had never been tested for HIV. One person seroconverted during the period when the project was being implemented in FCJ, which warrants a strategy of routinely testing persons returning to jail after an interval of >3 months. The cost per new diagnosis in this project is lower than the cost incurred in many screening programs set in other venues (2).

What is already known on this topic?

Integrating human immunodeficiency virus (HIV) screening as a routine component of the intake medical evaluation process in jails located in communities with a high prevalence of HIV infection facilitates case finding of persons who do not regularly access HIV testing from community sources, and helps reduce the stigma of HIV testing, thereby increasing awareness of HIV status and diagnosis among highly stigmatized groups, especially black men who have sex with men.

What is added by this report?

An HIV screening demonstration project conducted at the Fulton County Jail in Atlanta, Georgia, during 2011–2012 identified 0.4% of all tested jail entrants with newly diagnosed HIV infection, all of whom were provided medical care in the facility and referred to community services on release. Without this HIV screening project, these persons likely would have been diagnosed later in the course of their infection, resulting in delayed access to care and treatment, and possible transmission of HIV to their partners.

What are the implications for public health practice?

Public health administrators might consider collaborating with jail administrators to incorporate routine, opt-out HIV screening into the intake medical evaluation process of jails in communities with a high prevalence of HIV. In addition, because jail stays typically are short, linkage to community services is critical. An opportunity exists for the public health system and community-based organizations to collaborate with jails to ensure access to HIV care and retention in treatment for persons with HIV upon their release from jail.

Black men who have sex with men (MSM) are disproportionately infected with HIV, and an estimated 59% of black MSM are unaware of their infection (7). Nearly 40% of the black men newly diagnosed with HIV in this project reported sex with men. Making HIV screening a routine, rather than an exceptional, part of the medical evaluation process in jails in high HIV-prevalence, inner-city communities might help to decrease the stigma of HIV testing in jails and ultimately could decrease the number of persons in all risk categories who are unaware of their infection. There is no evidence of a disproportionate rate of incarceration among MSM compared with other men; however, minority populations, particularly blacks and Hispanics, are disproportionately incarcerated compared with whites (1). Hence, the integration of opt-out HIV screening into the intake process might decrease the number of black MSM who are unaware of their infection.

A study of routine, jail-based HIV testing conducted during 2000–2007 in Rhode Island revealed that 0.17% of tests resulted in new diagnoses (8). Although the number of newly diagnosed cases at Rhode Island’s jail declined during this observation period to 10 cases per year, the Rhode Island correctional HIV testing program was responsible for identifying 15% of all new HIV diagnoses in Rhode Island during the period. Rhode Island has one jail for the entire state; Georgia has more than 150 jails. However, the new HIV cases found at FCJ, where 41 of the 52 new cases of HIV were identified during 2011, represented approximately 5.4% (41 of 759) of all new HIV cases linked to Fulton County addresses and approximately 1.1% (41 of 3,621) of cases diagnosed in the state of Georgia that year (Jane M. Kelly; National Center for HIV/AIDS, Viral Hepatitis, STD, and TB Prevention, CDC; personal communication; 2013). Four additional large jails in the Atlanta metropolitan statistical area have average daily censuses of approximately 2,000–3,500 detainees (1), and none routinely screen for HIV during the medical intake evaluation. Routine, opt-out HIV testing in each of the other jails, if each had a similar rate of cases, might have identified an additional 164 persons in the Atlanta metropolitan statistical area in 2011.

The findings in this report are subject to at least three limitations. First, cases might have been misclassified as new if they previously had been diagnosed in another state and the patients failed to disclose their previous diagnosis to FCJ staff; only the state and local registry was checked. Second, the mean CD4 count at diagnosis might have been higher or lower than the value reported because the majority of newly diagnosed persons left jail before a CD4 count was obtained. Finally, the percentage of newly diagnosed persons who subsequently were linked to care after release is unknown; however, a previous demonstration project suggests that with adequate case management, a substantial percentage of these persons access care in the community (3).

The FCJ HIV screening project demonstrated that when a large jail in a high-prevalence community incorporated routine, opt-out HIV screening into the intake medical evaluation process, screening resulted in the diagnosis of persons previously unaware of their HIV infection. However, because of the very short detention period for most inmates, detainees with newly diagnosed HIV infection might be released before completion of pretreatment evaluation and initiation of HIV therapy. Linkage to community services is critical, and an opportunity exists for the public health system and community-based organizations to collaborate with jails to ensure access to HIV care and retention in treatment for persons with HIV released from jail (9,10). Research is needed to determine whether screening this population reduces transmission and prolongs survival, and whether interventions to increase linkage to community services are cost-effective.

## Figures and Tables

**TABLE:** Demographic and clinical characteristics of persons newly diagnosed with HIV upon entry to Fulton County Jail — Atlanta, Georgia, 2011–2012

	Male (n = 47)		Female (n = 5)	
		
Characteristic	No.	(%)	No.	(%)
**Mean age (yrs) (SD)**	33.7 (10.7)		33.3 (12.2)	
**Black race**	47	(100.0)	5	(100)
**Sexual behavior**				
Heterosexual sex	29	(61.7)	5	(100)
Men having sex with men	14	(29.8)	NA	—
Male and female partners	4	(8.5)	ND	—
**Documented narcotics use**	40	(85.1)	4	(80)
**Previous HIV test**				
Never tested	14	(29.8)	2	(40)
Ever tested for HIV	33	(70.2)	3	(60)
**Calendar years since most recent HIV test**				
1	8	(24.2)	—	—
2	13	(39.4)	1	(33)
3	9	(27.3)	—	—
4	3	(9.1)	2	(67)
**Any CD4 count in jail**	21	(44.7)	1	(20)
**Mean first CD4 (cells/mm** **^3^** **) (SD)**	372 (250)		374	
Range	31–950			
<200	4	—	0	—
200–349	7	—	0	—
350–499	5	—	1	—
≥500	5	—	0	—

**Abbreviations:** HIV = human immunodeficiency virus; SD = standard deviation; NA = not applicable; ND = not determined.

## References

[1] Minton TD (2011). Jail inmates at midyear 2010—statistical tables.

[2] Spaulding A, Reid L, Bowden C A tale of one city, two venues: comparing costs of routine rapid HIV testing in a high-volume jail and a high-volume emergency department, Atlanta, Georgia. Abstract no. 1061. http://www.retroconference.org/2013b/pdfs/1061.pdf.

[3] de Voux A, Spaulding AC, Beckwith C (2012). Early identification of HIV: empirical support for jail-based screening. PLoSOne.

[4] Office of National AIDS Policy (2010). National HIV/AIDS strategy for the United States.

[5] Hall HI, Holtgrave DR, Maulsby C (2012). HIV transmission rates from persons living with HIV who are aware and unaware of their infection. AIDS.

[6] CDC (2006). Revised recommendations for HIV testing of adults, adolescents, and pregnant women in health-care settings. MMWR.

[7] CDC (2010). Prevalence and awareness of HIV infection among men who have sex with men—21 cities, United States, 2008. MMWR.

[8] CDC (2010). Routine jail-based HIV testing—Rhode Island, 2000–2007. MMWR.

[9] CDC (2011). Vital signs: HIV prevention through care and treatment—United States. MMWR.

[10] CDC (2009). HIV testing implementation guidance for correctional settings.

